# Effects of Sedation by Intramuscular Administration of Medetomidine on Canine Abdominal Vascular System and Hepatic Parenchyma Imaging Using Enhancement Dynamic Computed Tomography

**DOI:** 10.3390/vetsci7030091

**Published:** 2020-07-13

**Authors:** Kenji Kutara, Teppei Kanda, Noritaka Maeta, Yohei Mochizuki, Yoshiki Itoh, Fumiko Ono, Taketoshi Asanuma

**Affiliations:** Faculty of Veterinary Medicine, Okayama University of Science, 1–3 Ikoinooka, Imabari, Ehime 794–8555, Japan; t-kanda@vet.ous.ac.jp (T.K.); n-maeta@vet.ous.ac.jp (N.M.); y-mochizuki@vet.ous.ac.jp (Y.M.); y-itoh@vet.ous.ac.jp (Y.I.); f-ono@vet.ous.ac.jp (F.O.); t-asanuma@vet.ous.ac.jp (T.A.)

**Keywords:** contrast enhanced computed tomography, medetomidine, contrast media, cardiovascular system, dog

## Abstract

This prospective crossover study compared the effects of intramuscular administration of medetomidine for sedation on parameters of the abdominal vascular system, measured by enhancement computed tomography (CT), to those of propofol-induced sevoflurane maintenance anesthesia, as a control, in five clinically healthy adult male beagle dogs (11.4–12.8 kg). Each animal underwent both protocols at a 1-week interval. The enhancement (HU) and time to peak enhancement on CT were measured for the aorta (AO), caudal vena cava (CVC), portal vein (PV), and hepatic parenchyma (HP). The contrast effects in the AO, PV, and HP were significantly delayed under medetomidine sedation compared to the control anesthesia protocol. Particularly, the contrast effect in the PV and HP was significantly delayed under sedation, appearing approximately 1 min after contrast medium injection. This delay likely reflects the peripheral vasoconstrictive effect of medetomidine. We noted a generally early high contrast enhancement of the CVC under medetomidine sedation, likely contributed by the induced bradycardia. Therefore, findings obtained on contrast enhancement CT under medetomidine sedation may be different from those obtained under propofol-induced sevoflurane maintenance anesthesia. These differences are important to consider when using the findings to inform diagnosis.

## 1. Introduction

In veterinary medicine, multiphasic multi-detector computed tomography (CT) angiography, including triple-phase helical CT, is currently used as a diagnostic tool for vascular disorders, such as portosystemic shunts [1], arteriovenous fistulae [2], and various tumors [3,4,5,6,7,8]. This method is also commonly used for characterizing parenchymal disease, such as pancreatic disease [9,10,11,12]. For triple-phase helical CT, a single bolus injection of contrast medium enhances imaging during the phase of preferential arterial enhancement, followed by the portal venous phase, and the delayed phase [5,6]. However, contrast enhancement of the vascular system by CT imaging can be influenced by various factors, including the injection protocol, the dose of iodine used, the formulation of the contrast medium, and the size of the catheter used [13,14,15].

CT imaging further requires that the animal be immobilized, with anesthesia or sedation used to achieve this [1,2,3,4,5,6,7,8,9,10,11,12,13,14,15,16,17]. However, agents used for anesthesia and sedation can influence the cardiovascular circulation. As an example, medetomidine, which is a highly selective α_2_-agonist that is widely used for sedation, produces cardiovascular effects, including vasoconstriction, a change in blood pressure, and bradycardia [18,19,20]. In humans, it is known that changes in cardiac output affect the timeline of contrast enhancement on CT angiography [21,22]. The effects of different methods of anesthesia and sedation on contrast enhancement CT angiography, however, have not been investigated in animals. Hence, the purpose of our study was to evaluate the effect of intramuscular (IM) medetomidine sedation on contrast enhancement of the abdominal vascular system, namely, the aorta (AO), caudal vena cava (CVC), and portal vein (PV), and on the hepatic parenchyma (HP) in dogs, compared to those of propofol-induced sevoflurane maintenance anesthesia as a control.

## 2. Materials and Methods

This was a prospective, randomized crossover study. The study protocol strictly followed the Rules for Animal Care and Use of the Laboratory Animal Center, Imabari Campus, Okayama University of Science, and was compliant with the Guide for the Care and Use of Laboratory Animals (8th ed). Experimental protocols were approved by the Animal Care and Use Committee of Okayama University of Science (approval number, 2019-04).

Five adult male beagle dogs (11.4–12.8 kg) were used in this study. These animals had not been used for any prior research. The dogs were housed in our Laboratory Animal Center at Imabari Campus, Okayama University of Science, under a 12:12 h light-dark cycle (light period, 8:00 a.m. to 20:00 p.m.). The room temperature was maintained between 24 and 26 °C and the humidity between 40–60%. For inclusion, all dogs had to be clinically healthy, based on a physical examination, complete blood count, and results from biochemical testing, abdominal radiography, and echocardiography. Needle catheters (22 G) were placed in the right or left cephalic vein for propofol injection and contrast medium infusion, with the side randomly allocated between animals before induction of anesthesia and sedation. After this study, all dogs were maintained in our Laboratory Animal Center.

### 2.1. Anesthesia and Sedation Protocols

Whether anesthesia or sedation was used first was randomly determined for each dog. The animals were fasted for approximately 12 h before anesthesia or sedation. For the control anesthesia protocol, dogs were induced using propofol (dose, 6.0 mg/kg), administered intravenously (IV), and intubated using a tracheal tube. General anesthesia was maintained using sevoflurane (2.5%: vaporizer setting) and oxygen (2 L/min) under mechanical ventilation (rebreathing type: respiratory rate, 10 breaths/min; respiratory pressure, 10 to 15 cmH_2_O; and end expiratory carbon dioxide concentration, 40 mmHg). For the sedation protocol, a 40 µg/kg dose of medetomidine hydrochloride was administered IM in the right semitendinosus or semimembranosus muscle. Spontaneous breathing was maintained under sedation, with no requirement for intubation; flow-by oxygen (100%) was provided (5 L/min). Each animal underwent both the anesthesia and sedation protocols at a 1-week interval. The vaporizer was calibrated before each experiment.

### 2.2. CT Imaging Protocol

Dynamic CT was performed using a 16-slice multidetector CT scanner (Aquilion Lightning, Canon Medical Systems, Otawara, Japan). All dogs were placed in the dorsal recumbent position. The CT scan was started after immobilization was stable (the scanning start time after injection propofol or medetomidine; approximately 20–30 min). The scanning position was localized at the level of T13. For the anesthesia protocol, the dynamic scanning parameters were as follows: rotation time, 1 s; slice thickness, 1 mm; reconstruction interval, 1 s; X-ray tube potential, 120 kV; and X-ray tube current, 100 mA; with a total scanning time of 60 s (60 slices). For the sedation protocol, the dynamic scanning parameters were as follows: rotation time, 1 s; slice thickness, 1 mm; reconstruction interval, 2 s; X-ray tube potential, 120 kV; and X-ray tube current, 100 mA; with a total scanning time of 120 s (60 slices). A single CT scan was performed at 2, 3, 4, and 5 min after the start of the contrast medium infusion for all sessions. The parameters for this single scan were as follows: rotation time, 1 s; slice thickness, 1 mm; X-ray tube potential, 120 kV; and X-ray tube current, 100 mA.

A non-ionic, low osmolar iodinated contrast material, Iohexol (Omnipaque 300; Daiichi-Sankyo Inc., Tokyo, Japan) was used, administered at a dose of 2 mL/kg (600 mgL/kg) via injection into the cephalic vein, using a power injector. The injection time was 15 s and the dynamic scan was initiated at the time the injection of the contrast medium. During scanning, heart rate was measured at 1-min intervals, while blood pressure was measured and recorded at two time-points, before anesthesia or sedation, and at the start of the scanning. Heart rate was measured by counting the QRS complex on the electrocardiogram over a 60 s period. Blood pressure was non-invasively measured using the oscillometric method with a multiparameter monitor (BSM-3592, Nihon Kohden Corporation, Tokyo, Japan), with the size of the blood pressure cuff selected to be about 40% of the limb circumference, applied to the forelimb (radius) for all measurements.

### 2.3. Image Analysis

**Figure 1:**
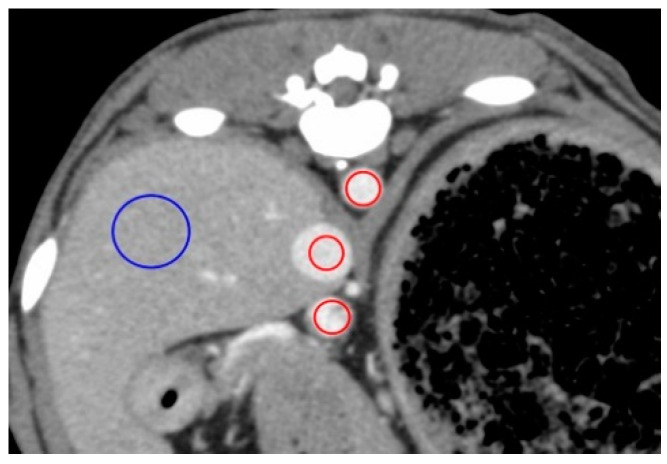
Measurement of the contrast values for the hepatic vessels and hepatic parenchyma on a representative image obtained 50 s after injection for the anesthesia protocol. The contrast values of the aorta (AO), caudal vena cava (CVC), portal vein (PV), and hepatic parenchyma (HP) were measured by placing the region of interest (ROI) in the center of the AO, center of the CVC, the center of the PV, and right lateral lobar parenchyma, respectively. ROIs demarcated by red lines have an area of 40 mm^2^ and those in blue have an area of 200 mm^2^.

All CT images were reviewed on a dedicated DICOM viewer (OsiriX MD, Pixmeo SARL., Bernex, Switzerland). Contrast enhancement measures were obtained at the following regions of interest (ROIs; Figure 1): 40 mm^2^ area at the center of the AO, PV, and CVC, and a 200 mm^2^ area in either the left of right lateral lobe of the liver for HP measures, avoiding the hepatic vessels. All measures in the regions of interest (ROIs) were recorded in Hounsfield units (HU). The following time-based parameters were also measured from the time of contrast medium injection: first appearance of AO enhancement (AO-EA) and the AO enhancement peak (AO-EP); first appearance of CVC enhancement (CVC-EA) and the CVC enhancement peak (CVC-EP); first appearance of PV enhancement (PV-EA) and the PV enhancement peak (PV-EP); and first appearance of HP enhancement (HP-EA) and HP enhancement peak (HP-EP). The time-attenuation curve (TAC) was calculated based on all measured values in the ROIs.

### 2.4. Statistical Analysis

Statistical analyses were performed using commercially available statistical analysis software (Stat Mate III, ATMs. Co, Tokyo, Japan). All ROI measurements and time-based parameters were reported as a median and range. The normality of the distribution of data was confirmed prior to analyses. The Kruskal–Wallis H test was used to evaluate the relationships among all measured values and time-based parameters, as well as body weight, heart rate, blood pressure, the injection time, and the speed of the contrast medium. A Mann–Whitney U test was used to evaluate the contrast values between the anesthesia and sedation protocols, as well as for between-group comparison of blood pressure before anesthesia or sedation and the time to the start of scanning. For all statistical analyses, a *p*-value < 0.05 was considered significant.

## 3. Results

**Figure 2:**
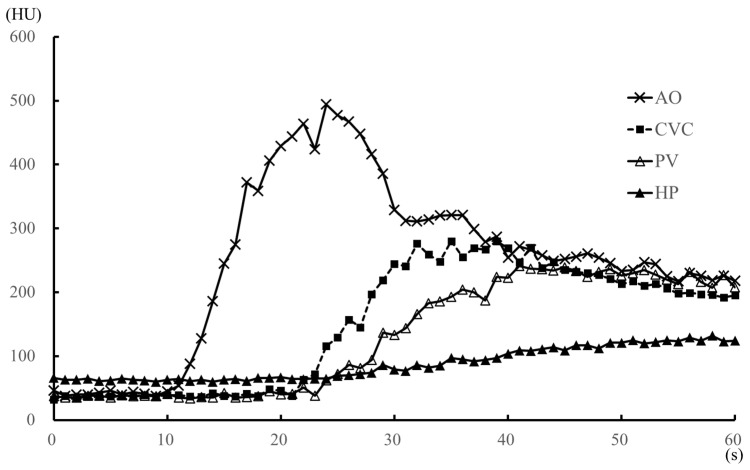
Median values of the time-attenuation curve (TAC) for the anesthesia (propofol and sevoflurane) protocol within 1 min after injection of the contrast medium, in five dogs. The TAC was generated from the median contrast values of the aorta, caudal vena cava, portal vein, and hepatic parenchyma. AO, aorta; CVC, caudal vena cava; HP, hepatic parenchyma; HU, Hounsfield unit; PV, portal vein.

**Figure 3:**
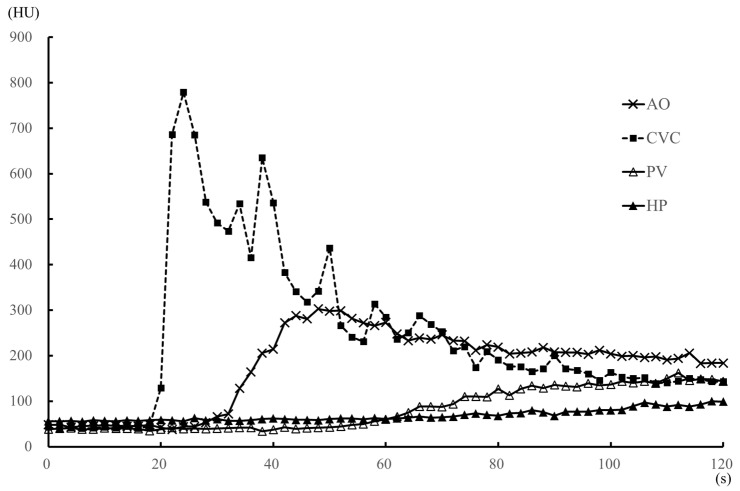
Median values of the time-attenuation curve (TAC) for the medetomidine sedation (medetomidine) protocol within 2 min after injection of the contrast medium, in five dogs. The TAC was generated from the median contrast values of the aorta (AO), caudal vena cava (CVC), portal vein (PV) and hepatic parenchyma (HP). The enhancement of the CVC was earlier than that of the AO, with a strong CVC enhancement peak.

All measurements obtained are summarized in Table 1, Table 2, Table 3 and Table 4. The TACs for the anesthesia and sedation protocols are shown in Figure 2 and Figure 3, respectively. With the sedation protocol, all dogs exhibited bradycardia (Table 3). Within each protocol, there were no differences with regard to ROI measurement values, time-based parameters, body weight, injection volume, and the speed of the contrast medium. With the anesthesia protocol, there was no characteristic change in heart rate (Table 3), although all blood pressure values were significantly lower at the start time of scanning than before anesthesia (Table 4). With the sedation protocol, heart rate was significantly lower at all time-points after sedation onset (Table 3), although there was no characteristic change in all blood pressure values (Table 4).

**Figure 4:**
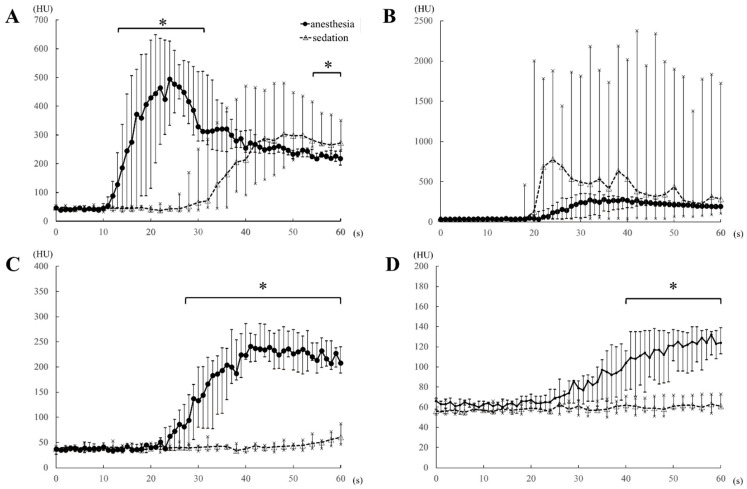
Comparison of the time-attenuation curve of the aorta (**A**), caudal vena cava (**B**), portal vein (**C**), and hepatic parenchyma (**D**) between the anesthesia (propofol and sevoflurane) and sedation (medetomidine) protocols, obtained within 1 min of the injection of the contrast medium, in five dogs. HU, Hounsfield unit; *, *p*-values < 0.05.

Compared to the sedation protocol, all time-based parameters (AO-EA, AO-EP, PV-EA, PV-EP, HP-EA, and HP-EP) were earlier for the anesthesia protocol (Table 1), while peak enhancement values for AO, PV, HP (Table 1) and the heart rate at each 1-min interval (Table 3) were significantly higher for the anesthesia protocol. However, all blood pressure values were significantly higher under the sedation than the anesthesia protocol (Table 4). Under anesthesia, AO contrast values were significantly higher than those under sedation during the 14–32 s after injection of the contrast medium, with the values at 54–60 s after injection being significantly lower (Figure 4A). There was no significant difference in AO contrast values at all other time points (Table 2 and Figure 4A). Observed differences in contrast values of the CVC were not significantly different between the two protocols at all time-points of measurement (Figure 4B). PV contrast values were significantly higher for the anesthesia than the sedation protocol in the early time period, 26 s after the injection of the contrast medium (Figure 4C), with no significant difference between the two protocols at ≥2 min after contrast medium injection (Table 2). Similarly, HP values were significantly higher under anesthesia than sedation in the early period, 40 s after the injection of the contrast medium (Figure 4D), with no significant difference between the two protocols at ≥3 min after contrast medium injection (Table 2).

**Figure 5:**
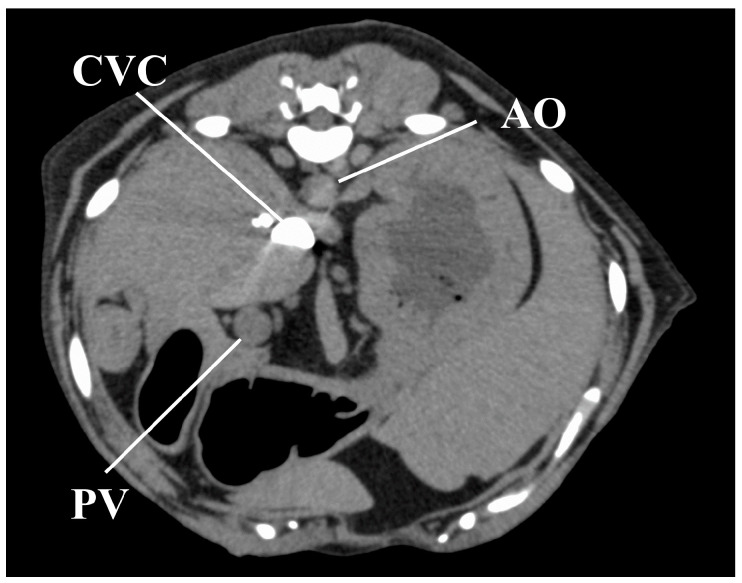
Image obtained 11 s after injection of the contrast medium in one representative dog under the sedation protocol, showing a strong CVC enhancement, with no contrast in the AO and PV. AO, aorta; CVC, caudal vena cava; PV, portal vein.

With regard to time-based parameters, in four dogs under the sedation protocol, CVC enhancement occurred earlier than AO enhancement, with a strong peak of CVC enhancement (Figure 5). We noted that in one dog in the sedation protocol, an early strong CVC enhancement was not observed, with a CVC-EA value of 50 s, CVC-EP value of 74 s, and a peak enhancement value of 154 HU (Table 1). Not only was the contrast enhancement earlier with the sedation than the anesthesia protocol (namely, CVC-EA and CVC-EP) in this animal, but also the peak enhancement value of CVC (CVC-EP) reached a maximum under the sedation protocol. All dogs fully recovered from anesthesia and sedation.

## 4. Discussion

In veterinary medicine, the effects of contrast medium in abdominal vessels have been widely investigated [13,14,15]. These studies have used anesthesia during imaging, including the use of propofol, isoflurane, and sevoflurane. It is for this reason that we compared the effects of sedation using IM medetomidine on the abdominal vascular system to those obtained under inhalant anesthesia. In this study, the median values of AO-EP and PV-EP were 24 s and 37 s, respectively. The time of enhancement peak in AO and PV were comparable to those reported in previous studies [13,14,15], confirming the validity of using the anesthesia protocol as the control.

The rotation time and total time of dynamic scanning were 1 s and 60 s, respectively, under anesthesia and 2 s and 120 s, respectively, under sedation. Of note, under anesthesia, the effects of the contrast medium appeared early (i.e., within 1 min after contrast medium injection) and were tightly coupled, as previously reported [13,14,15]. Thus, the rotation time of 1 s and the total scanning time of 60 s were appropriate and reduced overall radiation exposure. We do note, however, that in our clinical experience, the contrast enhancement of the artery and portal vein was not observed during the arterial phase (15–20 s after the start of the contrast medium injection) and the portal venous phase (40 s after the start of the injection), respectively, in triple phase helical CT when sedation, including medetomidine, was used. Hence, we set an increase in the rotation time of 2 s and an extension in the total scanning time of 120 s to capture all contrast effects in the abdominal vasculature in sedation protocol. The setting of a rotation time of 1 s and the total scanning time of 120 s was not possible with the specification of the CT scanner that we used in this study. Our findings, therefore, confirmed differences in measured effects of contrast enhancement between the anesthesia and the sedation protocols. Specifically, under anesthesia, the contrast effects in the abdominal vasculature and HP, as well as peak enhancement effects, all appeared within 1 min after the injection of the contrast medium, which is consistent with previously reported time-based parameters of the effects of contrast enhancement [13,14,15]. By comparison, under sedation, early contrast enhancement effects, within 1 min of injection of the contrast medium, occurred only in the AO and CVC, with effects being earlier in the CVC than AO, except in one dog. The appearance and peak enhancement effects in the PV and HP occurred at a time lag >1 min after the injection of the contrast medium. Overall, all measured enhancement parameters (AO-EA, AO-EP, PV-EA, PV-EP, HP-EA, and HP-EP) occurred earlier under anesthesia than sedation.

In humans, the timing of contrast effects is affected by cardiac output [21,22]. Consistent with previous findings of an association between medetomidine and bradycardia [18], all dogs in our experiment exhibited bradycardia under the sedation. In the previous studies, the intramuscular administration of 40 µg/kg medetomidine induced significant bradycardia in dogs, in agreement with our findings [23,24]. The medetomidine induced bradycardia and vasoconstriction would result in a decrease in cardiac output [25], with the severe decrease in cardiac output explaining the approximately 20-s delay in the AO-EA and AO-EP with sedation compared to the anesthesia protocol. The approximately 1-min delay in PV-EA, PV-EP, HP-EA, and HP-EP between the sedation and anesthesia protocols may be related to the vasoconstriction effects of medetomidine [18,19,20]. The PV is a component of the venous system, collecting blood from the abdominal peripheral artery [26], with most of the blood supply to the HP being dependent on the PV [27]. As such, we propose that the large delay in the contrast effect in the PV and HP compared to that in the AO with the sedation protocol might be caused by medetomidine-induced peripheral vasoconstriction, as well as by bradycardia. We do note, however, that we did not measure vasoconstriction in our study protocol.

With sedation, except in one dog, contrast enhancement of the CVC appeared earlier than the effect in the AO. Moreover, contrast enhancement values of the CVC were the highest among all measured contrast enhancement values, with this state persisting for at least 40 s after the administration of contrast medium. This finding is indicative of a sedation-induced bradycardia and a weakened cardiac output; therefore, the contrast medium may have stagnated in the CVC without entering the pulmonary circulation. In humans, this characteristic effect is known as the “inferior vena cava level contrast” or “dependent pooling sign” and is observed clinically in patients who are in shock due to cardiac arrest, cardiac tamponade, or myocardial infarction [28,29,30]. This effect, in humans, results from the contrast medium permeating into the CVC from the right atrium due to reduced cardiac output and blood pressure. However, all dogs in our study did not exhibit hypotension under sedation. Hence, the CVC effect observed was likely due to a reduced cardiac output secondary to both, bradycardia and an increase in afterload.

In veterinary medicine, CT angiography, including dual-phase and triple-phase helical CT, is widely used in clinical and experimental fields [1,2,3,4,5,6,7,8,9,10,11,12]. In these contrast CT imaging protocols, the timing of the start of scanning was determined based on previously reported TACs [13,14,15]. The arterial phase and portal venous phase are generally scanned within 1 min of the injection of the contrast medium: arterial phase, 15–20 s, and the portal venous phase, 30–40 s after injection. The delayed phase occurs about 2 min after contrast medium injection [1,2,3,4,5,6,7,8,9,10,11,12]. Under the sedation protocol in our study, the AO-EA appeared approximately 30 s after contrast medium injection and the peak AO enhancement about 1 min after injection. PV enhancement was delayed to about 1 min after contrast medium injection, with PV-EP attained between 1.5 and 3 min after the injection. As well, compared to the anesthesia protocol, peak contrast values for the AO, PV, and HP were significantly lower for the sedation protocol. Therefore, with medetomidine sedation, the contrast effects for the target vascular would not be observed if the CT scan protocol was performed using usual timing of arterial phase and portal phase. This means that, in addition, the contrast enhancement patterns for CT angiography previously described [1,2,3,4,5,6,7,8,9,10,11,12] could not be applied to images obtained under IM medetomidine sedation.

The limitations of our study need to be acknowledged. Foremost, with regard to cardiac parameters, we only measured heart rate and blood pressure. As such, the underlying cause of observed differences in contrast enhancement effects with medetomidine sedation could only be inferred. A detailed examination of circulation parameters, including cardiac parameters, is necessary to clarify the mechanisms underlying the observed effects of medetomidine sedation on contrast enhancement. We also note that while we used propofol and sevoflurane for the anesthesia protocol and medetomidine for the sedation protocol, in practice, various opioid and sedative agents (such as α2, acepromazine, or a benzodiazepine) are widely used as premedication for general anesthesia. These agents do exert a dose- and drug-dependent effect on the cardiovascular system and, therefore, could have an impact on the results of CT angiography. We did not use such premedication drugs in our study and, as such, cannot predict their effect on measured outcomes under IM medetomidine sedation. Further studies are needed to investigate the influence of other sedative and anesthetic agents on measured contrast effects during CT angiography.

## 5. Conclusions

In conclusion, our results demonstrate that contrast enhancement measured under IM medetomidine sedation was greatly delayed compared to measures under anesthesia. In addition, peak contrast effects in the AO, PV, and HP were significantly lower under sedation than under anesthesia. Of note was the early high contrast enhancement of the CVC under sedation. Therefore, findings on CT angiography will be very different under medetomidine sedation from those obtained under anesthesia. These differences need to be considered when using contrast enhancement findings to inform diagnosis.

## Figures and Tables

**Table 1:** Comparison of the enhancement appearance and peak times, as well as the peak contrast values of all vessels and liver parenchyma between the anesthesia (propofol and sevoflurane) and sedation (medetomidine) protocols in five dogs.

Parameters	Anesthesia Protocol	Sedation Protocol	*p*-Value
AO–EA (s) *	13 (12–18)	30 (26–38)	0.012
AO–EP (s) *	24 (21–31)	48 (40–58)	0.013
Peak contrast value of AO (HU) *	558.3 (494.7–648.3)	343.1 (289.2–481.7)	0.012
CVC–EA (s)	21 (20–32)	20 (18–50)	0.526
CVC–EP (s)	37 (34–45)	40 (24–74)	1
Peak contrast value of CVC (HU)	313.5 (228.1–381.4)	779.6 (154.2–2375.6)	0.144
PV–EA (s) *	25 (24–30)	64 (54–78)	0.012
PV–EP (s) *	43 (41–44)	104 (90–180)	0.011
Peak contrast value of PV (HU) *	244.4 (236.2–285.9)	166.3 (162.1–199.3)	0.012
HP–EA (s) *	25 (18–35)	50 (42–94)	0.012
HP–EP (s) *	56 (51–60)	240 (108–240)	0.012
Peak contrast value of HP (HU) *	132.4 (127.5–132.8)	111.4 (106.7–125.2)	0.012

Note. The data shown are median values with ranges. AO, aorta; AO-EA, Arrival time to the AO enhancement appearance; AO-EP, arrival time to the AO enhancement peak; CVC, caudal vena cava; CVC-EA, Arrival time to the CVC enhancement appearance; CVC-EP, Arrival time to the CVC enhancement peak; HP, hepatic parenchyma; HP-EA, Arrival time to the HP enhancement appearance; HP-EP, Arrival time to the HP enhancement peak; HU, Hounsfield Unit; PV, portal vein; PV-EA, Arrival time of PV enhancement appearance; PV-EP, Arrival time to PV enhancement peak; s, second; *, significantly different between anesthesia protocol and sedation protocol (*p*-values < 0.05).

**Table 2:** Comparison of change in the contrast values every minute between the anesthesia (propofol and sevoflurane) and sedation (medetomidine) protocols in five dogs.

Parameters	0 min	1 min	2 min	3 min	4 min	5 min
the contrast values of AO; anesthesia protocol (HU)	46.1 (42.8–48.9)	218.7 (195.8–244.6)	174.6 (157.3–199.8)	141.2 (129.8–184.6)	127.4 (119.7–158.2)	122.2 (110.5–147.5)
the contrast values of AO; sedation protocol (HU)	48.2 (47.4–55.3)	273.6 (247.2–351.4)	184.3 (144.3–232.1)	146.3 (137.6–157.8)	131.6 (109.2–133.8)	123.4 (98.3–141.1)
*p* value	0.073	0.012 *	0.835	0.463	0.833	0.834
the contrast values of CVC; anesthesia protocol (HU)	32.8 (30.9–41.5)	195.4 (177.7–215.7)	159.5 (142.0–184.3)	131.5 (109.8–159.1)	122.1 (113.5–145.2)	111.9 (100.9–127.6)
the contrast values of CVC; sedation protocol (HU)	43.3 (34.1–45.2)	285.3 (104.4–1722.1)	143.5 (118.2–357.0)	117.6 (88.8–144.7)	108.5 (94.4–114.4)	100.6 (81.9–104.4)
*p* value	0.142	0.675	0.296	0.296	0.356	0.096
the contrast values of PV; anesthesia protocol (HU)	37.6 (27.3–29.4)	208.1 (188.8–240.0)	175.4 (161.2–216.7)	139.4 (127.4–187.1)	125.5 (117.4–161.4)	118.5 (112.1–137.4)
the contrast values of PV; sedation protocol (HU)	38.2 (37.4–42.8)	60.3 (46.2–87.6)	144.5 (142.6–178.9)	142.4 (123.3–166.4)	137.8 (118.4–147.2)	122.0 (99.7–136.8)
*p* value	0.142	0.012 *	0.142	1	0.531	0.676
the contrast values of HP; anesthesia protocol (HU)	66.2 (58.4–69.1)	124.8 (113.1–139.0)	122.2 (111.7–129.4)	111.1 (102.0–114.8)	107.6 (96.5–111.7)	98.4 (93.3–113.8)
the contrast values of HP; sedation protocol (HU)	56.1 (53.2–67.4)	61.2 (60.0–78.1)	99.3 (77.1–112.0)	107.8 (104.7–120.2)	111.4 (98.4–125.1)	110.4 (97.8–119.6)
*p* value	0.143	0.012 *	0.021 *	0.917	0.142	0.173

Note. The data shown are median with ranges. AO, aorta; CVC, caudal vena cava; HP, hepatic parenchyma; HU, Hounsfield Units; min, minute; PV, portal vein; *, *p*–values < 0.05.

**Table 3:** Comparison of median heart rate between the anesthesia (propofol and sevoflurane) and sedation (medetomidine) protocols in five dogs.

Group	Heart Rate before Procedure	Heart Rate of 0 min (bpm)	Heart Rate of 1 min (bpm)	Heart Rate of 2 min (bpm)	Heart Rate of 3 min (bpm)	Heart Rate of 4 min (bpm)	Heart Rate of 5 min (bpm)
anesthesia protocol	100 (140–83)	103 (78–136)	101 (76–131)	102 (79–126)	101 (73–127)	103 (71–124)	103 (68–122)
sedation protocol	108 (138–85)	44 (40–48) **	40 (36–44) **	36 (28–44) **	44 (36–48) **	40 (36–48) **	44 (36–44) **
*p*-value	1	0.012 *	0.012 *	0.012 *	0.012 *	0.011 *	0.011 *

*, *p*-values < 0.05; **, *p*-values < 0.05 compared to the heart rate before the procedure (0 min: 0.023, 1 min: 0.004, 2 min: 0.004, 3 min: 0.005, 4 min: 0.005, 5 min: 0.004 for the sedation protocol).

**Table 4:** Comparison of median blood pressure between the anesthesia (propofol and sevoflurane) and sedation (medetomidine) protocols in five dogs.

Group	The Point of before Protocol Procedure			The Point of Start of Scan		
	SAP (mmHg)	DAP (mmHg)	MAP (mmHg)	SAP (mmHg)	DAP (mmHg)	MAP (mmHg)
anesthesia protocol	160 (138–212)	110 (109–138)	124 (106–148)	91 (88–108) **	50 (38–62) **	64 (62–84) **
sedation protocol	156 (134–170)	94 (88–129)	107 (100–138)	147 (129–170)	101 (96–124)	124 (111–144)
*p*-value	0.835	0.593	0.403	0.012 *	0.012 *	0.012 *

Note. The data shown are medians with ranges. bpm, beat per minute; DAP, diastolic arterial pressure; MAP, mean arterial pressure; SAP, systolic arterial pressure, *, *p*-values < 0.05, **, *p*-values < 0.05 compared to the blood pressure before the procedure (MAP: 0.011, DAP: 0.002 MAP: 0.001 in anesthesia protocol).
